# Th1 Platform Immune Responses Against *Leishmania major *Induced by Thiol-Specific Antioxidant-Based DNA Vaccines

**DOI:** 10.5812/jjm.8974

**Published:** 2014-02-01

**Authors:** Fatemeh Tabatabaie, Mehdi Mahdavi, Sobhan Faezi, Abdolhossein Dalimi, Zohreh Sharifi, Lame Akhlaghi, Fatemeh Ghaffarifar

**Affiliations:** 1 Department of Parasitology and Mycology, School of Medicine, Iran University of Medical Sciences (IUMS), Tehran, IR Iran; 2 Department of Virology, Pasteur Institute of Iran, Tehran, IR Iran; 3 Department of Bacteriology, Pasteur Institute of Iran, Tehran, IR Iran; 4 Department of Parasitology, Faculty of Medical Sciences, Tarbiat Modares University, Tehran, IR Iran; 5 Blood Transfusion Research Center, High Institute for Research and Education in Transfusion Medicine, Tehran, IR Iran

**Keywords:** DNA vaccine, *Leishmania major*, Thiol-S Antioxidant, Aluminum Phosphate

## Abstract

**Background:**

The Thiol-specific antioxidant (TSA) is an antigen of *Leishmania major* which is believed to be the most promising molecule as a vaccine candidate against leishmaniasis.

**Objectives:**

In this study, we investigated the protective efficacy of TSA-based DNA vaccine against *L. major* infection.

**Materials and Methods:**

Recombinant plasmid construction TSA (pcTSA) was prepared and transfected into eukaryotic cells and expression was confirmed with western blot and RT-PCR. The mice were assigned to six different groups and DNA immunization was performed with 100 µg intramuscular recombinant plasmid with a two-week interval. Cytokines and lymphocyte proliferation assay, antibody responses and determination of parasite burden were performed following immunization and the challenging infection with *L. major*.

**Results:**

The antibody and IFN-γ titers were higher in pcTSA + AlPO4 group the immunized mice with pcTSA alone, but there was no statistically significant difference between the two groups. Additionally the IL-4 titer was not statistically different between the groups following immunization and challenge. After infection with *L. major* promastigotes, the immunized mice with pcTSA and the one immunized with both pcTSA + AlPO4 presented a considerable reduction in diameter of lesion but there was no statistical difference between the two groups. The immunized mice had significantly lower parasite loads. No significant differences were observed between the two vaccinated groups. However the highest reduction in parasite burden was observed in the group immunized with pcDNA + AlPO4. No significant differences were observed in survival rate of the immunized mice after the challenge with *L. major*.

**Conclusions:**

In conclusion, TSA-based DNA vaccine induced Th1 platform immune response and aluminum phosphate could improve the efficacy of these vaccines with induction of humoral and cellular immune responses against *L. major* infection. There were no significant differences observed between pcTSA and pcTSA + AlPO4 groups.

## 1. Background

Leishmaniasis is a parasitic disease caused by several species of the genus *Leishmania*. It is a prevalent disease in many parts of the world with about 12 million infected cases. There are 1.5-2 million new cases of cutaneous and 500,000 cases of visceral leishmaniasis reported annually (1-3). Treatment of leishmaniasis is a complicated process due to the toxicity, and side effects of the available drugs and resistance against them. Resistant variant cases of both cutaneous and visceral leishmaniasis have become more common and re-infection occurs rapidly (4, 5). Development of either new anti-*Leishmania* drugs or a vaccine seems to be vital. Immunity against re-infection is acquired following cutaneous infection. Unfortunately no protective and effective anti-*Leishmania* vaccine is available at the moment in spite of several tested vaccine protocols. Among the vaccine candidates, TSA (Thiol-specific antioxidant protein) has been introduced as one of the most predominant vaccine candidates (6-8). The *L. major* TSA protein is homologous to eukaryotic TSA with molecular weight of 22.1 KDa, which is composed of 200 amino acids and placed on chromosome 15. On the other hand the TSA is expressed in the *L. major* promastigote and amastigote (9). 

In the recent years, a number of strategies to potentiate DNA vaccines have been studied, ranging from antigen-targeting to viral vectors, liposomes or microparticles, etc. A number of these strategies have been able to considerably provoke the immune system, however the use of DNA vaccines as vaccine adjuvant has shown some limitations (10). Aluminum salts have been extensively and most commonly used as adjuvants for commercial human vaccines, mostly because of their correlation with a large variety of protein antigens, outstanding safety and low cost. AlPO_4_ triggers humoral immune responses and slightly supports the generation of specific IFN-γ producing CD8^+^ T cells (11). Aluminum phosphate seems to keep or even increase the Th bias of the immune response induced by DNA vaccines, which makes it a very suitable candidate to be used as an adjuvant for vaccines against intracellular parasite *Leishmania* (12, 13). 

## 2. Objectives

In this study, we investigated the protective efficacy of TSA-based DNA vaccine against *L. major* infection. Here we have shown that TSA DNA–vaccine stimulated high titers of specific antibody, high levels of IFN-γ and lymphocyte proliferation and low levels of IL-4 and phenotypic markers of Th1 immune responses, which are required for the control of this parasite (1, 3, 14).

## 3. Materials and Methods

### 3.1. Preparation of L.major -TSA DNA Vaccine and Its Transfection Into Eukaryotic Cells

In a previous study, we cloned and transfected recombinant pcTSA into eukaryotic cells (15). Briefly, the amplified TSA coding sequence by PCR from the genomic DNA of *L. major* strain MHRO/IR/75/ER was cloned into the polylinker of plasmid pTZ57R/T (Ferments). The recombinant plasmid DNA was purified from the transformed *Escherichia coli* and confirmed by restriction analysis. The TSA gene was cloned with linkers to join to the HindIII and EcoRI sites of the eukaryotic expression vector, pcDNA3 (Invitrogen, USA), to produce a recombinant pcTSA vector and finally sequencing was performed. For *in vitro* transfection, the CHO (Chinese hamster ovary) cells were grown in Dulbecco’s Modified Eagle’s Medium (DMEM, Gibco), which was supplemented with 100 U mL^-1 ^penicillin and streptomycin and 10 % fetal calf serum (FCS). Transfection was performed with a transfection kit (Genejuice Transfection Kit, Novagen, USA), according to the manufacturer’s instructions.

### 3.2. SDS-PAGE and Western Blot Analysis

In the previous study, recombinant protein expression was confirmed by SDS-PAGE and immunoblot methods (16). Briefly, the cells (transfected and non-transfected as controls) were harvested and lysed in a sample buffer, 72 hours following the transfection. After sonication and freeze-thawing, the cells were concentrated by centrifugation and their protein profile was used for SDS-PAGE and western blot analysis (16). The recombinant *L. major* TSA protein was expressed and separated by SDS-PAGE and transferred into a nitrocellulose membrane. For the immunoblot assay, the membranes were blocked overnight and sequentially probed with Leishmania antibody-positive mice sera or anti His-tag antibody (1/2000 dilution) (Qiagen, USA) and an anti-mouse IgG horseradish-peroxidase (Sigma, USA); specific binding was revealed with diaminobenzidine (DAB) (DAKO, Denmark). 

### 3.3. Antigen Preparation

Soluble *Leishmania *antigen was prepared from stationary phase promastigotes of *L. major* after a few passages in the Schneider’s medium. About 2 × 10^6^* L. major* promastigotes were washed five times in cold sterile phosphate-buffered saline (PBS) containing 1 mM phenylmethanesulfonyl fluoride (PMSF). After five freeze-thaw cycles, the suspension was centrifuged. The supernatant was then collected, dialyzed and quantified by the Bradford assay (17), filtrated in a 0.2 μm pore filter (Nalge Company, USA), and finally stored at – 80 °C. 

### 3.4. Mice Immunization and Experimental Infection 

Six to eight week-old BALB/c female mice were purchased from Razi Institute (Karaj, Iran) and maintained under standard conventional conditions. The mice were divided into 6 different groups, each containing 5 mice receiving different regimens of DNA immunization. Aluminum phosphate was used for preparation of TSA plasmid vaccine formulation. The process was carried out according to the method of Bradford et al., by which kinetic of adsorption was determined after mixing 100 μg of pcTSA plasmid with 45 μg of aluminum phosphate in 100 μL of PBS solution for each injection (18).

The mice were grouped based on the administration content described below:

Group 1: PBS (control group)
Group 2: AlPO_4_ (control group)
Group 3: pcDNA3 (control group)
Group 4: pcDNA3 + AlPO_4_ (control group)
Group 5: pcTSA (vaccinated group)
Group 6: pcTSA + AlPO_4_ (vaccinated group)

The mice in each group were anesthetized with a 25 μL/g mixture of 10% Ketamine and 2% Xylazine injected intraperitoneally (IP) and immunized via intramuscular (IM) injection into both quadricepses with 100 μL of administration content according to their grouping. They were not injected more than 50 μL per muscle (18). Three inoculations were employed with the same DNA and AlPO_4_ doses and the same immunization schedule was applied at two-week intervals. 

Two weeks after the last immunization, the mice of each group (immunized and control mice) were challenged at the base of tail through the intradermal (ID) route with 2 × 10^6^ promastigotes of *L. major* (strain MHRO/IR/75/ER). Measurement of diameters of the lesion at the site of inoculation was performed weekly by a Vernier caliper thereafter. Then, the animals were sacrificed and the spleens and serum samples were harvested for immunological purposes. 

### 3.5. Lymphocyte Proliferation Assay

Two weeks after the third immunization, the spleens were taken from immunized mice under sterile conditions, dissected and resuspended in sterile cold PBS, containing 2% FBS. RBCs were disrupted with lysis buffer and the single-cell suspension was adjusted to 2×10^6^ cells/mL with supplemented RPMI 1640 (Gibco, Germany). The diluted cell suspensions (100 µL/well) were dispensed into 96-well flat-bottom culture plates (Immunlon, Dynatech, USA) and stimulated with 20 µg/ml of soluble *Leishmania *antigen (SLA) from *L. major *as an antigen recall. Phytohemagglutinin-A (5 μg/mL, Gibco), unstimulated wells and complete culture medium were used as positive, negative and blank controls, respectively.

 After 72 hours of culture, 20 µL of 3[4,5-dimethylthiazol-2-ll]-2,5-diphenyltetrazolium bromide; thiazolyl-blue (MTT) was added to each well and incubation continued for another 4 hours. Following incubation, the supernatant from each well was aspirated and solubilization of formazan crystals was carried out by adding 100 µl of dimethyl sulfoxide. The absorbance of each well was determined at 540 nm. Proliferation activity was reported as stimulation index (SI) which was calculated according to the following formula: OD _540 nm_ of stimulated wells/OD _540 nm_ of un-stimulated wells. 

### 3.6. Cytokines Assay

Before and after the challenge with *L. major,* cytokine assays were performed. Briefly, splenocytes from immunized mice were plated in duplicate in 24-well plates (MaxiSorb, Nunc, Denmark). The cells were then stimulated with soluble *Leishmania *antigen (SLA) from *L. major *(20 µg/mL), at 37 °C in 5% CO_2_ for 72 hours. The culture supernatants were harvested for the cytokine assay. The levels of gamma interferon (IFN-γ) and IL-4 were assessed by sandwich enzyme-linked immunosorbent assay (DuoSet ELISA Development Kit, USA), according to the manufacturer’s instructions. 

### 3.7. Antibody Assay

The sera were tested for the presence of specific anti-*L. major *IgG antibodies by an optimized indirect ELISA. First, the blood samples were collected (n = 5/group) from the immunized mice two weeks after the final booster injection and seven weeks after the infection. The samples were centrifuged for serum preparation. The 96-well microtiter plates (MaxiSorb, Nunc, Denmark) were coated with soluble *L. major* antigens (10 µg/mL) in 100 mM carbonate-bicarbonate buffer (pH 9.6) (100 μL/well) and incubated overnight at 4 °C. The plates were washed with PBS buffer.

Blocking was carried out with 5% dried skimmed milk in PBS buffer for 1 hour at 37 °C. After washing with PBS containing 0.05% Tween 20 (PBST20), 1:200 diluted serum samples in 1% BSA-PBST20 were added to each well and incubated at 37^ º^C for 1.5 hours and washed 4 times after the reaction. Then, 100 µL of 1:5000 diluted peroxidase-conjugated anti-mouse IgG antibody (DAKO, Denmark) was added and incubated at 37 ^º^C for 1 hour and then washed 4 times. The peroxidase activity was developed by Tetra methyl benzydine (TMB) substrate. The reaction was stopped by adding H_2_SO_4_ (2 M) and the optical density (OD) was read at 450 nm in an ELISA micro plate reader (Bio-Rad, USA). 

### 3.8. Determination of Parasite Burden and Lesion Diameter 

In order to determine the parasite burden in experimental mice, seven days after the final immunization, mice (n = 3 group) were sacrificed and their spleen was removed. An equal piece of spleen from each mouse was excised and homogenized using a tissue grinder in 2 mL of the Schneider’s Drosophila medium supplemented with 20% heat-inactivated FBS and Gentamicin (0.1%). Afterwards, serial dilutions in the range of 1 to 1.4 × 10^-4^ were prepared and cultured in 96-well plates at 26 °C. Seven to fifteen days after culture, the plates were monitored with an inverted microscope (40X magnification). The presence of mobile promastigotes in each well was recorded. The last dilution for which the wells contained at least one parasite was reported as the final titer and the number of parasite per gram of tissue was calculated using the following formula: Parasite burden = - log_10 _(parasite dilution/tissue weight) (8, 19). Diameter of the lesions was also measured by a Vernier caliper after challenge. 

### 3.9. Statistical Analysis

Statistical comparisons between the experimental groups were carried out with an analysis of variance (ANOVA) and Tukey’s (SPSS V17) Post-hoc test. Statistical analysis of survival time was carried out with the Kaplan-Meier (Log-Rank) test for five mice in each group. Differences were statistically considered significant when *P *values were less than 0.05 (*P* < 0.05).

## 4. Results

### 4.1. Construction of L. major TSA DNA Vaccine

#### 4.1.1. Presence of TSA Gene in the DNA Vaccine and Protein Confirmation

In the previous study, we cloned the TSA gene into pTZ57R/T vector. This recombinant plasmid was extracted from transformed *E. coli* (TG1 strain) and sequenced (15). In another study, the recombinant vector expressed and produced the TSA protein in the eukaryotic expression system. Briefly, the recombinant vector containing the TSA gene was transfected and expressed in eukaryotic cells (CHO cells) and finally confirmed by SDS-PAGE and immunoblot analysis (16).

### 4.2. Lymphocyte Proliferation Assay

**Figure 1.**
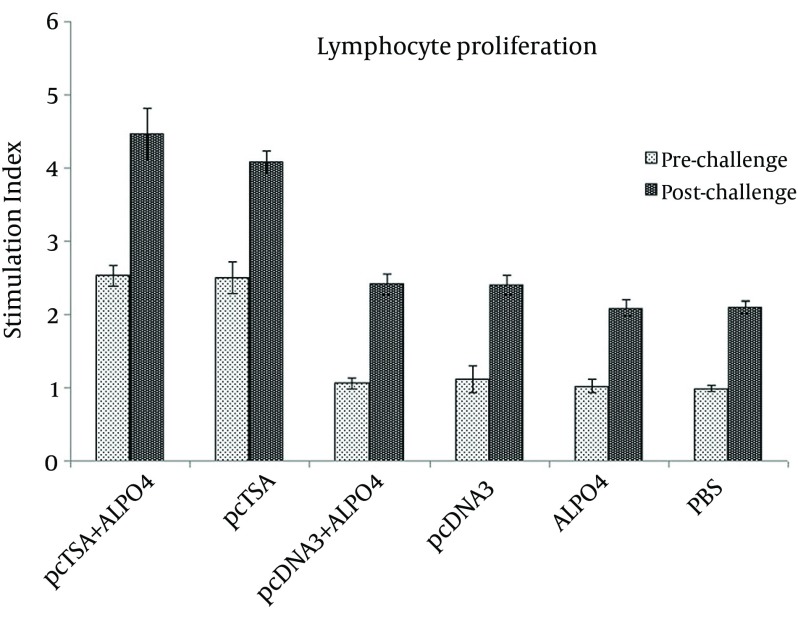
Lymphocyte Proliferation of Experimental Groups After Immunization Periods Splenocytes of mice were harvested and stimulated in vitro for three days. Proliferation was presented as stimulation index of individual mice and data is presented as mean of triplicate ± standard deviation (SD).

To further investigate the phenotype (Th1/Th2 pattern) of the immune response elicited by immunization of SLA, suspensions of spleen cells from immunized mice and control groups were isolated and re-stimulated in vitro with 20 µg/mL of SLA. As shown in Figure 1, mice immunized and co-immunized with pcTSA and pcTSA + AlPO4 (group 5 and 6), induced a much better specific proliferation response, in comparison with the control groups (P < 0.05). This shows the ability of pcTSA and pcTSA + AlPO4 to induce spleen cell proliferation as a marker of cellular immune responses, whereas no significant difference were observed between pcTSA and pcTSA + AlPO4 (P > 0.05). The same results were obtained after the challenge, so that the immunized mice in pcTSA and pcTSA + AlPO_4_ groups showed significantly increased lymphocyte proliferation compared to the control groups (P < 0.05) (P < 0.05).

### 4.3. Cytokine Release Assays

**Figure 2.**
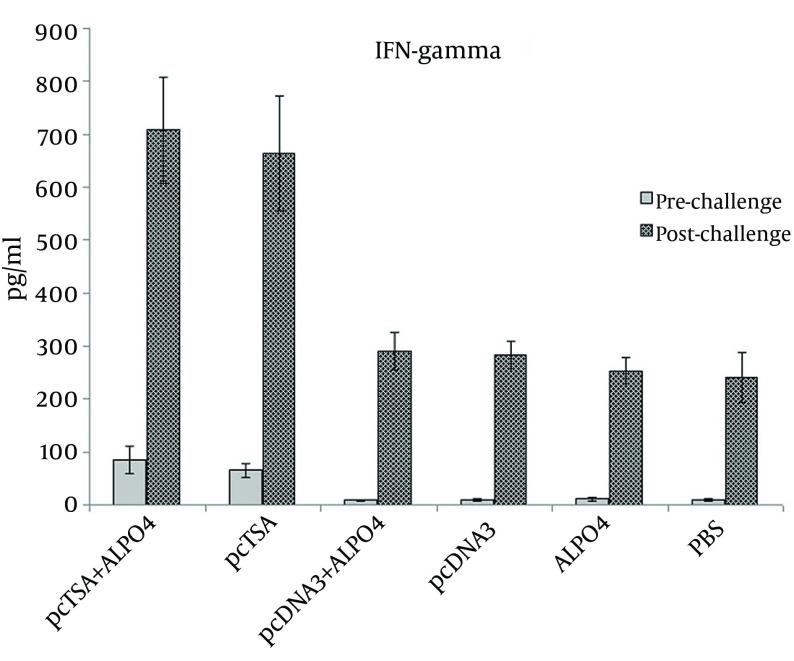
ELISA Assays on The Culture Supernatants Showed High Levels of IFN-γ. Cytokine production (IFN-γ) by the splenocytes of the vaccinated mice at three weeks after the last vaccination and seven weeks after the challenge infection.

**Figure 3.**
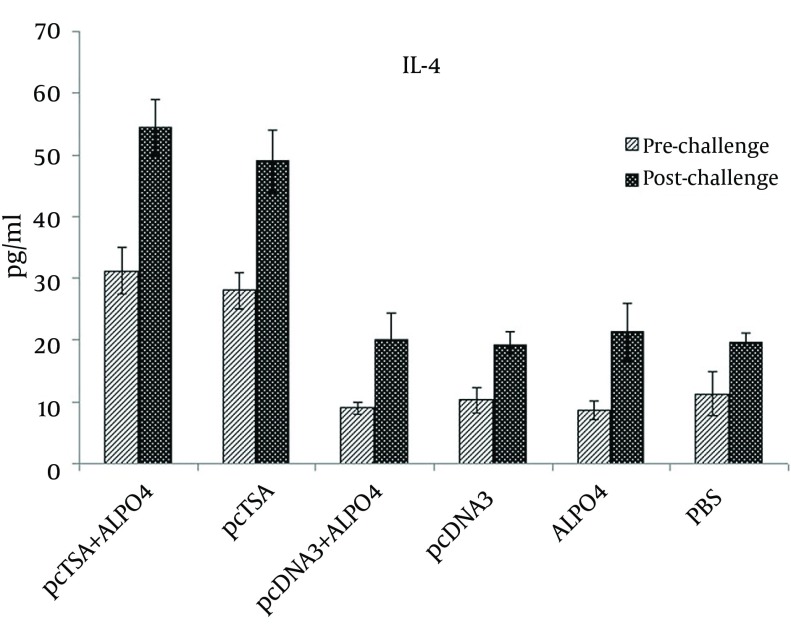
Cytokine Production (IL-4) by the Splenocytes of the BALB/c Mice Immunized With TSA DNA and pcTSA+ AlPO_4_ at Three Weeks After the Last Vaccination and Seven Weeks After the Challenge Infection.

The IFN-γ and IL-4 cytokines production in the supernatants of spleen cells of experimental groups was evaluated after the final booster injection and seven weeks after the challenge infection with *L. major*. The pcTSA and pcTSA + AlPO_4_ groups showed a sustained IFN-γ production following immunization and after the challenge infection (Figure 2). IFN-γ values markedly increased in the pcTSA and pcTSA + AlPO_4_ groups, which were significantly higher than in the control groups (P < 0.05). Although, in the immunized mice with pcTSA + AlPO_4 _elicited IFN-γ values were greater than the immunized mice with pcTSA alone before and after the challenge infection, yet, there was no statistically significant difference between the two groups (P > 0.05). IL-4 values of the spleen cells from the immunized and control (for all the groups) mice were slightly increased before and after the challenge infection, however, there were no significant differences between these groups (P > 0.05) before and after the challenge with *L. major* (Figure 3). 

### 4.4. Total Specific IgG ELISA

**Figure 4.**
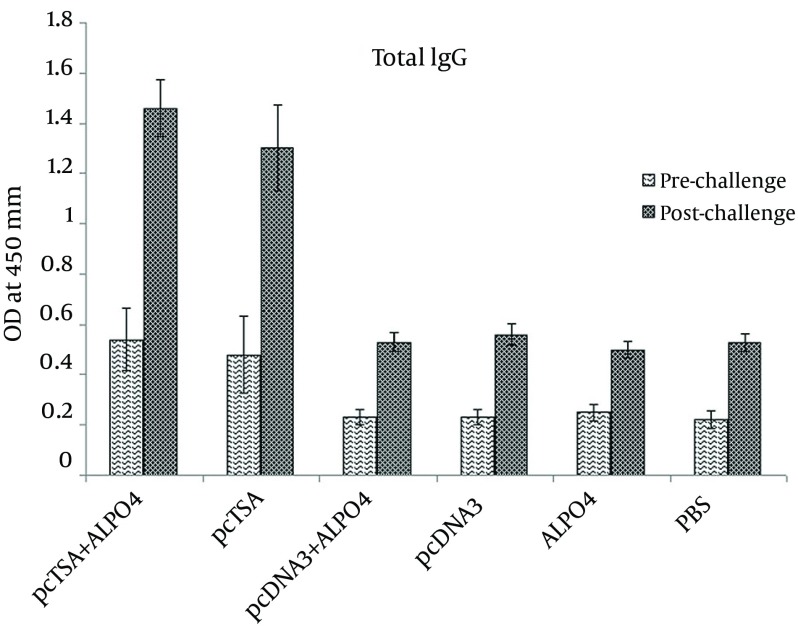
The Effect of Aluminum Phosphate on Humoral Protection (IgG) Against *L. major* Infection Induced by DNA Vaccination Three Weeks After the Last Vaccination and Seven Weeks After the Challenge Infection. Sera of individual mice were collected and specific total IgG level was evaluated with an optimized indirect ELISA method as mentioned in the materials and methods section.

The sera were collected from the immunized and control group mice 2 weeks after the final booster injection and seven weeks after the challenge infection and tested for the presence of total specific IgG antibodies by the indirect ELISA method. As shown in Figure 4, the antibody responses were elicited by the immunization before & after the challenge infection. Anti *L. major* IgG values markedly increased in the pcTSA and pcTSA + AlPO_4_ groups which were significantly higher than the control groups. Although pcTSA + AlPO_4_ elicited IgG antibody values were greater than in the sera of the mice immunized with pcTSA alone before and after the challenge infection, there was no statistically significant difference between the two groups (P > 0.05).

### 4.5. Determination of Parasite Burden

**Figure 5.**
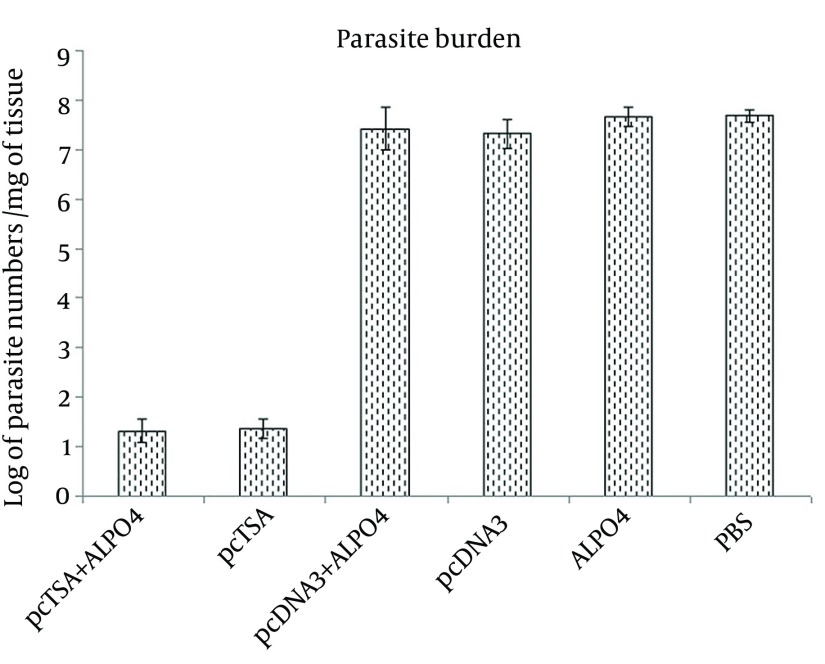
Parasite Quantitation in the Spleen Cells of the Challenged BALB/c Mice Seven Weeks After Immunization by Intradermal Inoculation of 2 × 10^6^ Promastigotes of *L. major* in the Base of Tail. Parasite burden in the spleen was calculated as –log_10 _(parasite dilution/tissue weight).

Result of parasite burden in the spleens of experimental groups showed that, the immunized mice had significantly lower parasite loads compared to the mice in the control group (P < 0.05). No significant differences were observed between the two vaccinated groups (P > 0.05), although the highest reduction in parasite burden was observed in immunized mice with pcTSA + AlPO_4_ (Figure 5). 

### 4.6. Lesion Development and Survival Rate

Lesion development was followed by weakly measurements using a Vernier caliper. Kinetics of lesion evaluation in BALB/c mice showed that following infection with 2 × 10^6 ^*L. major* promastigotes, the immunized mice with the DNA vaccine alone and DNA vaccine + AlPO_4_ presented a noticeable reduction in diameter of lesion and an increaseof weight compared to the control mice (P < 0.05).

**Figure 6.**
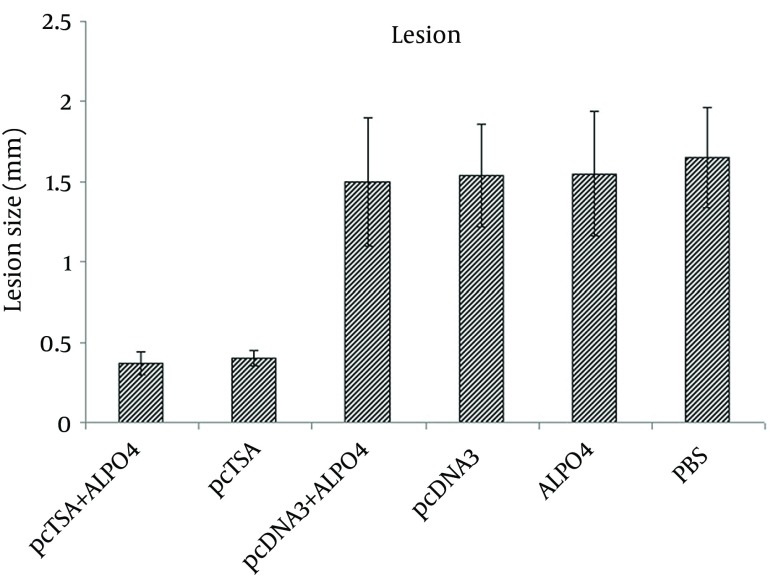
Lesion Size (Diameter of Dermal Lesion) and Weight Increase in the Challenged BALB/c Mice Seven Weeks After the Immunization by Intradermal Inoculation of 2 × 10^6^ Promastigotes of *L. major* in the Base of the Tail.

The immunized mice with the DNA vaccine + AlPO_4_ showed a greater reduction in diameter of lesion, compared to the DNA vaccine alone but there was no statistically significant difference between these two groups (P > 0.05) (Figure 1). All mice in the control groups died within 13 weeks after the challenge. The survival time of the immunized mice with pcTSA and pcTSA + AlPO_4_ was significantly higher than the control groups (P < 0.05) after the challenge with *L. major* but there was no statistical difference between the immunized groups (P > 0.05) (Figure 6). 

## 5. Discussion

In view of the emerging drug resistance, the development of safe and efficient vaccines remains to be the best hope for achieving definitive control of leishmaniasis (6). Immunity against re-infection is acquired following cutaneous infection with *Leishmania* spp., suggesting that prophylactic immunization is feasible (5, 14). In the recent years, significant progress has been made in the identification of vaccine candidates inducing a protective response (20-22). A number of vaccine strategies have been tested, ranging from killed parasites to recombinant antigens or DNA vaccines (5, 14, 23, 24).

The most successful vaccine attempts in humans have been achieved with whole-killed *Leishmania* promastigotes (whole-killed Leishmaniasis) with or without BCG. Unfortunately, the autoclaved parasite shows decreasing efficacy with time. Studies on the efficacy of thimerosal-preserved and non-autoclaved preparations have suggested a better lead to preservation. Vaccination of rhesus monkeys with heat-killed *Leishmania *promastigotes, using IL-12 and alum as adjuvants, has been shown to be safe and efficacious (25). 

In this study, we showed that immunization and co-immunization of mice with pcTSA and pcTSA + AlPO4 improves spleen cell proliferation and induces Th1-specific immune responses; manifested by the increased antigen-specific serum IFN-γ levels when compared with the DNA vaccine alone (group 3). Our results are comparable with Coler et al. findings which showed that administration of polyprotein vaccine enhances cellular immune responses. They showed that enhanced antigen-specific proliferative response increases IFN-γ production (26).

Several studies showed that the recombinant TSA protein with IL-12, induces excellent protection in the BALB/c mice (1, 14). These vaccines have not been proven to be able to induce complete and long lasting protection. DNA vaccination has become the fastest growing field in vaccine technology following reports at the beginning of the 90s, which suggested that plasmid DNA induces an immune response to the plasmid-encoded antigen. The CD8^+^ and CD4^+^ T-cells are efficiently induced by DNA vaccines via recruitment of both MHC-I and MHC-II pathways, while a soluble antigen, such as recombinant protein, generally induces only antibody responses.

In the present study, immunization of BALB/c mice with TSA DNA vaccine confers high levels of protective immunity (humoral and cellular). Studies show that TSA as a vaccine candidate for *Leishmania* could stimulate lymphocyte proliferation and Th1 cytokine profile (26). Our results also showed that TSA induces a predominant INF-γ production and low levels of IL-4, phenotypic markers of Th1 responses (1, 3, 14). *Leishmania* TSA protein is known as an antigenic compound in both murine and human systems and is constitutively expressed in both promastigote and amastigote life stages (2, 20, 24).

In attempting to develop a DNA vaccine, we focused on the gene encoding TSA, because previous studies for preventing leishmaniasis, have shown that immunization with TSA peptides, proteins or DNA vaccine can elicit a broad range of immune responses that are capable of decreasing infection in animals infected with *L. major*, yet it is necessary to increase their immunogenicity (14). Successful immunization that induces protection against leishmaniasis is highly dependent on adjuvant formulation. The formulation of plasmid DNA with certain mineral salts (for example AlPO_4_ that are the only adjuvants approved for general human and veterinary use) can enhance humoral immunity and the number and affinity of peptide antigen-specific T-cells secreting IFN-γ (27). Thus aluminum salts, particularly aluminum phosphate may be very practical adjuvants for the TSA DNA vaccines due to their low cost, easy use and safety record (12, 15, 23, 28). 

Different doses of DNA vaccines formulated with AlPO_4_ induced enhanced humoral responses and supported priming of MHC class I restricted cellular immunity (18, 23, 24, 28-30). In the present study, antibody responses, after immunization with pcTSA and pcTSA + AlPO_4_ before and after the challenge with *L. major* were increased and were significantly higher than that of the control groups. However, pcTSA + AlPO_4_ elicited IgG antibody values were greater than that in the sera of the mice immunized with pcTSA alone and there were no statistically significant differences between the two related groups before and after the challenge infection. This indicates that pcTSA was able to influence the immune response towards Th2 by inducing an IgG response. When AlPO_4_ is formulated with plasmid, anti-*L. major* IgG values increase more than those of plasmid alone and this study suggests that AlPO_4_ may represent an attractive alternative to increase the efficacy of this type of vaccines towards antibody production. 

The IFN-γ values were markedly increased in the pcTSA and pcTSA + AlPO_4_ groups, which were significantly higher than in the control groups following immunization and after the challenge with *L. major*. Although pcTSA + AlPO_4_ elicited IFN-γ values were greater than the immunized mice with pcTSA alone, there was no statistical difference between these two groups. IL-4 values were increased in all groups, but there were no statistical difference between these groups following immunization and post-challenge with *L. major*. 

Following infection with 2 × 10^6^* L. major* promastigotes, the immunized mice with pcTSA and pcTSA + AlPO_4_ presented a noticeable reduction in diameter of the lesion and an increaseof weight compared to the control mice. The immunized mice with the DNA vaccine plus aluminum phosphate were compared to those who received DNA vaccine alone, but there were no statistical differences between these groups. The number of parasites in the spleen was not significantly different between the unvaccinated mice. The immunized mice had significantly lower parasite loads compared to the control group mice. No significant differences were observed between the two vaccinated groups; however, the highest reduction in parasite burden was observed in the mice immunized with pcTSA + AlPO_4_. The survival time of the immunized mice with pcTSA and pcTSA + AlPO_4_ was significantly higher than the control groups after the challenge with *L. major*. 

Taken together, this study showed that the TSA-DNA vaccine formulated in alum with stimulation of cellular immune response could be an excellent candidate for further vaccine development against *Leishmania major.* However, there were no significant differences observed between the pcTSA and pcTSA + AlPO_4_ groups. 
